# Results and indications changes by prenatal diagnosis of 3,458 pregnant women: a 10-year retrospective study

**DOI:** 10.1080/07853890.2025.2563753

**Published:** 2025-09-22

**Authors:** Chao Wang, Haomiao Zhang, Yu Liu, Ruijing Wang, Qiao Li, Xianghan Zhang, Xin Huang, Yanhui Zhang, Meimei Liu

**Affiliations:** ^a^ Department of Obstetrics and Gynecology, The Second Affiliated Hospital of Harbin Medical University, Harbin, China; ^b^ Capital Medical University, Beijing, China

**Keywords:** Prenatal diagnostic indications, copy number variation sequencing, karyotype analysis

## Abstract

**Background:**

This study investigated changes in prenatal diagnostic indications among pregnant women in Heilongjiang Province, China, over a 10-year period (2014–2023) and evaluated the clinical efficacy of karyotype analysis and copy number variation sequencing (CNV-seq) for prenatal diagnosis.

**Method:**

We retrospectively analyzed data from pregnant women who underwent amniocentesis based on prenatal diagnostic criteria from January 2014 to June 2023 and provided consent, focusing on prenatal diagnostic indications, fetal chromosomal karyotype analyses, and CNV-seq results.

**Result:**

Among the 3,458 pregnant women, prenatal diagnosis indications included a high risk of NIPT (23.13%, 800/3,458) and a high risk of biochemical serological screening (22.30%, 771/3,458). Since 2017, the proportion of NIPT combined with fetal ultrasound abnormalities rose steadily, reaching 57.14% after 2020. Karyotype analysis was performed on 3,341 successfully cultured samples, revealing an abnormal karyotype detection rate of 18.25% (610/3,341). Structural chromosomal abnormalities accounted for 35.08% (214/610), followed by trisomy 21 (34.26%, 209/610) and sex chromosomal abnormalities (20%, 122/610). The overall positive predictive value (PPV) of NIPT was 44.40%, with a PPV for trisomy 21 of 81.72%. CNV-seq testing covered 2,771 samples, detecting pathogenic variations in 16.60% (460/2,771). Notably, 80 cases of pathogenic variation were identified in samples with normal karyotypes, and ​​14 cases were found in 108 samples without karyotyping. The highest pathogenic variation detection rate was observed in the NIPT high-risk group(42.21%, 320/758). Combining CNV-seq with other tests increased the anomaly detection rate to 20.36% (704/3,458), a 2.11% improvement over karyotype analysis alone.

**Conclusion:**

In recent 10 years, prenatal diagnosis indications in Heilongjiang Province have shifted to a "​​NIPT high-risk + abnormal ultrasound​​" pattern. Combined karyotype and CNV-seq testing​​ achieves a 20.36% anomaly detection rate. NIPT enables non-invasive screening​​ for fetal chromosome aneuploidy, while CNV-seq - a key technology for accurate prenatal diagnosis - helps reduce birth defects.

## Introduction

Birth defects (BDs) are a leading cause of early miscarriage, stillbirth, perinatal and infant mortality, and congenital disability. According to the WHO, globally, around 240,000 newborns die within 28 days of birth due to BDs. As the most populous country in the world, China has a high incidence of BDs. The ‘China Birth Defects Prevention and Control Report (2012)’ indicates a birth defect rate of ∼5.6%, with about 900,000 new cases annually, including 250,000 clinically apparent at birth [1]. Prenatal screening and diagnosis, using ultrasound, biochemical serum testing, and NIPT, are crucial for BDs secondary prevention [2]. Prenatal diagnosis typically involves invasive sampling methods like amniocentesis, chorionic villus sampling, or cordocentesis for fetal genetic testing. Despite chromosome karyotype analysis being the main lab method, Next-Generation Sequencing (NGS) technology has gained prominence due to its advantages, such as shorter reporting time, higher resolution, high detection flux, and lower DNA requirement. What’s more, the past decade has seen China’s fertility policy adjustments (the universal two-child policy in 2016 and three-child policy in 2021) and widespread NIPT adoption. In this context, the increase in older pregnant women has led to a change in the trend of prenatal diagnosis indications. The indication for prenatal diagnosis presents the characteristics of ‘high risk of NIPT + ultrasound abnormality’. Although studies [3] highlight CNV-seq’s advantages in microdeletion and microduplication syndrome detection, there is still a lack of decade-long, region-specific data comparing karyotype analysis and CNV-seq efficacy in China. Globally, ACOG’s 2020 guidelines recognize NIPT as a first-line screening tool for high-risk women (e.g. advanced maternal age, ultrasound abnormalities), with invasive diagnosis (e.g. CNV-seq) needed for confirmation [4]. The UK National Screening Council also recommends NIPT for medium-to-high-risk groups, with CNV-seq retesting to improve microdeletion syndrome detection [5]. This study focuses on Heilongjiang Province, an important medical center in Northeast China, with a prominent position in the application of traditional prenatal diagnostic techniques, and our practice is a response to global trends. Taking a single area as the research sample, the in-depth analysis of the situation in the past 10 years has unique research value and can effectively represent the characteristics of Heilongjiang region. It not only fills the gap in related research in this region for a long time, but also has a very important guiding significance for the regional prenatal screening and diagnosis service process and policy formulation in Heilongjiang region and even in the world.

## Methods

### Participant information

The study group comprised pregnant women who sought prenatal diagnosis at the Prenatal Diagnosis Center of the Second Affiliated Hospital of Harbin Medical University from January 2014 to June 2023. All participants voluntarily underwent prenatal diagnostic procedures voluntarily and provided written informed consent. The study was approved by the Medical Ethics Committee of the Second Affiliated Hospital of Harbin Medical University.

### Inclusion and exclusion criteria

The inclusion criteria are based on the current implementation document Technical Standards for Cytogenetic Diagnosis of Fetal Chromosome anomalies (2011).

The inclusion criteria for pregnant women were as follows: between 16^+0^ and 33^+6 ^weeks of gestation, including those older than 26 weeks who preferred amniocentesis to umbilical cord blood puncture for personal reasons; those who underwent amniocentesis based on identified prenatal diagnostic needs; individuals without contraindications to invasive prenatal diagnostic interventions; and participants who were able to undergo invasive puncture procedures and participate in follow-up. The exclusion criteria for individuals were as follows: who had contraindications to invasive puncture procedures (e.g. suspected preoperative infections, imminent miscarriage risks, active phases of systemic illnesses), who were unable to comply with procedural requirements, or who were not available for follow-up.

### Research methods

#### Group classification

Participants were classified into eight groups based on primary prenatal diagnostic indicators: fetal ultrasound anomalies (FUA), fetal structural abnormalities or abnormalities of more than 2 ultrasound soft indicators; high-risk biochemical serological screening (HBSS); high-risk NIPT (HNIPT); adverse family history (AFH), including one or both partners with chromosomal abnormalities; adverse obstetric history (AOH), including the birth history of children with chromosomal abnormalities, unexplained abortion history, etc.; advanced maternal age (AMA), age ≥35 years at the expected time of delivery; other indications not covered above; and multiple indications (MI), where two or more criteria were met. Patients with high-risk NIPT and other indications were categorized into the HNIPT group.

#### Invasive prenatal diagnostic procedures

A total of 3,458 participants underwent amniocentesis, in which 30 ml of amniotic fluid was extracted for analysis (23–25 ml for chromosomal karyotype analysis and 5–7 ml for CNV-seq).

#### Amniotic fluid karyotyping

Of these, 3,350 underwent karyotyping, involving standard procedures, such as centrifugation, culturing, fluid exchange, trypsin digestion, slide preparation, and staining. The analysis included counting 20 karyotypes per patient, or a minimum of 5 cells, with at least 50 metaphases counted in ambiguous cases.

#### Amniotic fluid CNV-seq

CNV-seq was performed on 2,771 samples, involving DNA extraction, library construction, library capture, sequencing on the BGISEQ2000 sequencer, and analysis using PSCC software, based on the GRCh37/hg19 reference genome.

### Statistical analysis

The data were analyzed using SPSS software, version 22.0. Prenatal diagnostic indications, the proportion of chromosomal abnormalities, and CNV-seq results are expressed as counts and percentages (%).

#### Reliability and deviation description of the data sources

The limitations of obtaining the data from a single center (Heilongjiang Provincial Prenatal Diagnosis Center) may limit the generalizability of the results, but such sample collection increases the reliability of the data.

## Results

### Distribution and composition ratio of indications for interventional prenatal diagnosis

Between 2014 and 2023, 3,458 pregnant women were included in this study. Among them, the main indication for prenatal diagnosis was HNIPT, accounting for 23.13% (800/3,458), followed by HBSS, 22.30% (771/3,458), and FUA, 21.66% (749/3,458). The fourth place was AMA, representing 10.38% (359/3,458). The lowest proportion was for AFH (86/3,458) and other factors (66/3,458) (Table 1).

**Table 1.** Distribution and composition ratios of prenatal diagnostic indications among 3,458 pregnants (cases, %).

Amniocentesis indication	2014	2015	2016	2017	2018	2019	2020	2021	2022	2023	Total
HBSS	123 (57.21)	131 (56.71)	38 (35.19)	68 (30.91)	131 (30.97)	113 (20.73)	47 (13.99)	73 (14.12)	27 (5.18)	20 (5.85)	771 (22.30)
HNIPT	10 (4.65)	9 (3.90)	10 (9.26)	61 (27.73)	94 (22.22)	142 (26.06)	83 (24.70)	139 (26.89)	158 (30.33)	94 (27.49)	800 (23.13)
FUA	10 (4.65)	17 (7.36)	14 (12.96)	21 (9.55)	76 (17.97)	132 (24.22)	109 (32.44)	129 (24.95)	157 (30.13)	84 (24.56)	749 (21.66)
AMA	30 (13.95)	39 (16.88)	11 (10.19)	20 (9.09)	49 (11.58)	41 (7.52)	33 (9.82)	67 (12.96)	38 (7.29)	31 (9.06)	359 (10.38)
AOH	10 (4.65)	11 (4.76)	12 (11.11)	18 (8.18)	25 (5.91)	51 (9.35)	30 (8.93)	29 (5.61)	42 (8.06)	28 (8.19)	256 (7.40)
AFH	6 (2.80)	5 (2.16)	4 (3.70)	7 (3.18)	4 (0.95)	11 (2.02)	8 (2.38)	18 (3.49)	13 (2.50)	10 (2.92)	86 (2.49)
Others	0 (0.00)	1 (0.04)	0 (0.00)	2 (0.09)	8 (1.89)	8 (1.47)	6 (1.79)	11 (2.13)	16 (3.07)	14 (4.09)	66 (1.91)
MI (≥2)	26 (12.09)	18 (7.79)	19 (17.59)	23 (10.45)	36 (8.51)	47 (8.62)	20 (5.95)	51 (9.86)	70 (13.43)	61 (17.84)	371 (10.73)
Total amniocentesis cases	215 (6.22)	231 (6.68)	108 (3.12)	220 (6.36)	423 (12.23)	545 (15.76)	336 (9.72)	517 (14.95)	521 (15.07)	342 (9.89)	3,458

HBSS: high-risk biochemical serological screening; HNIPT: high-risk NIPT; FUA: fetal ultrasound abnormalities; AMA: advanced maternal age; AOH: adverse obstetric history; AFH: adverse family history; MI: multiple indications.

**Figure 1.**
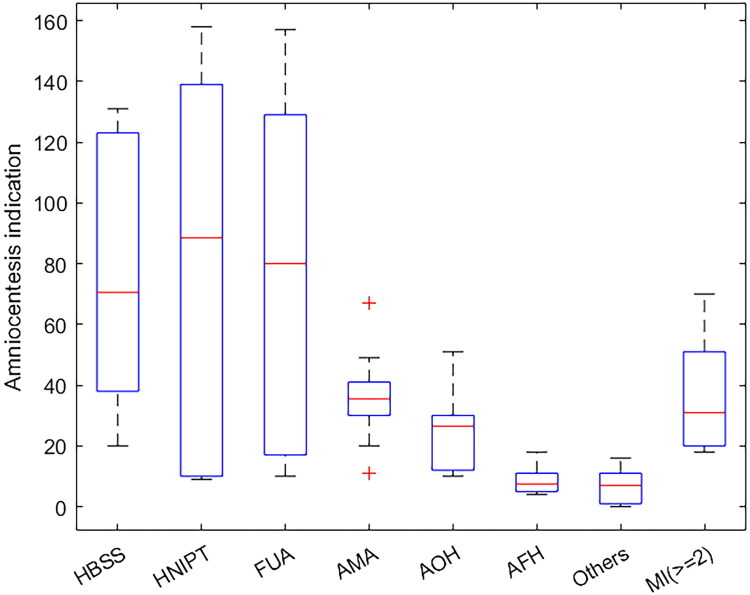
Indications for amniocentesis. HBSS: high-risk biochemical serological screening; HNIPT: high-risk NIPT; FUA: fetal ultrasound abnormalities; AMA: advanced maternal age; AOH: adverse obstetric history; AFH: adverse family history; MI: multiple indications.

In FUA group, the detection rate of chromosome abnormality in amniotic fluid was 21.66% (749/3,458). The proportion of HNIPT and FUA has been high since 2017. Although HBSS was the most common indication in the first 5 years (2014–2018), the proportion of HBSS has decreased year by year since 2018 as the proportion of HNIPT and FUA have gradually increased (Table 2, Figure 1).

**Table 2.** Ranking of prenatal diagnostic indication proportions.

Year	First	Second	Third	Fourth	Fifth
2014	HBSS (57.21%)	AMA (13.95%)	MI (≥2) (12.09%)	HNIPT; AOH; FUA (all 4.65%)	AFH (2.80%)
2015	HBSS (56.71%)	AMA (16.88%)	MI (≥2) (7.79%)	FUA (7.36%)	AOH (4.76%)
2016	HBSS (35.19%)	MI (≥2) (17.59%)	FUA (12.96%)	AOH (11.11%)	AMA (10.19%)
2017	HBSS (30.91%)	HNIPT (27.73%)	MI (≥2) (10.45%)	FUA (9.55%)	AMA (9.09%)
2018	HBSS (30.97%)	HNIPT (22.22%)	FUA (17.97%)	AMA (11.58%)	MI (≥2) (8.51%)
2019	HNIPT(26.06%)	FUA (24.22%)	HBSS (20.73%)	AOH (9.35%)	MI (≥2) (8.62%)
2020	FUA (32.44%)	HNIPT (24.70%)	HBSS (13.99%)	AMA (9.82%)	AOH (8.93%)
2021	HNIPT (26.89%)	FUA (24.95%)	HBSS (14.12%)	AMA (12.96%)	MI (≥2) (9.86%)
2022	HNIPT (30.23%)	FUA (30.13%)	MI (≥2) (13.43%)	AOH (8.06%)	AMA (7.29%)
2023	HNIPT (27.49%)	FUA (24.56%)	MI (≥2) (17.84%)	AMA (9.06%)	AOH (8.19%)

HBSS: high-risk biochemical serological screening; HNIPT: high-risk NIPT; FUA: fetal ultrasound abnormalities; AMA: advanced maternal age; AOH: adverse obstetric history; AFH: adverse family history; MI: multiple indications.

**Figure 2.**
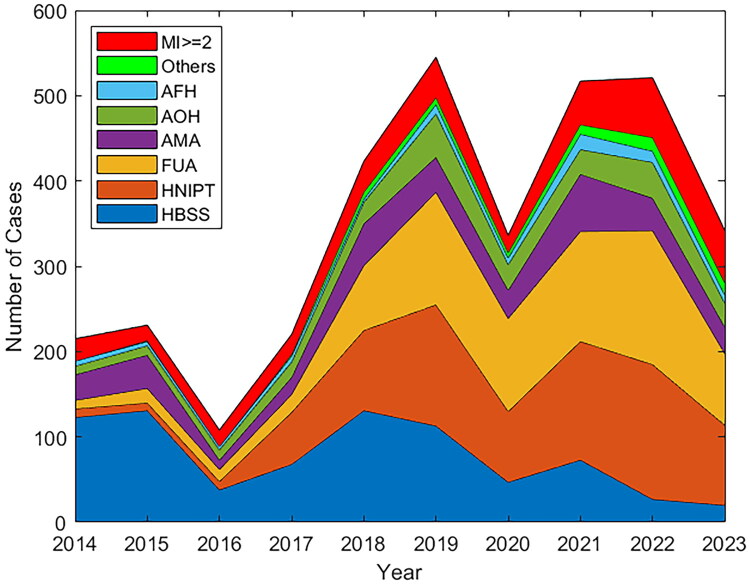
Trend of prenatal diagnostic indications in Heilongjiang Province (2014–2023) is displayed using stacked area plot. HBSS: high-risk biochemical serological screening; HNIPT: high-risk NIPT; FUA: fetal ultrasound abnormalities; AMA: advanced maternal age; AOH: adverse obstetric history; AFH: adverse family history; MI: multiple indications.

Over the past decade, prenatal diagnostic indications have undergone significant changes. The proportion of HBSS decreased from more than 50% in the early years to 5.85% in 2023. As a result of new screening techniques, HNIPT increased from 4.65% in 2014 to 30.33% in 2022, becoming one of the main indicators. The number of FUA cases has steadily increased in the past decade, with the composition ratio rising from 4.65% in 2014 to 32.44% in 2020, and then maintaining a high level. The composition ratio of AMA was also relatively stable. The number of AOH cases increased slowly in the early stage, but fluctuated in the later stage, and the composition ratio also changed accordingly. The number of AFH cases and the proportion of AFH components are relatively low, and there are fluctuations in the past 10 years, and there is no obvious dominant trend. The number of other puncture indications was small, but showed a slow upward trend, and the composition ratio increased slightly, indicating that some less clear factors gradually emerged in prenatal diagnosis. The number and composition ratio of multiple puncture indications (≥2) fluctuated and showed an increasing trend in the later period. In 2023, the composition ratio reached 17.84%, possibly due to more comprehensive assessment of pregnant women or an increase in complex risk factors (Figure 2).

### Detection rate of chromosomal abnormalities and the distribution of 610 chromosomal abnormalities in prediagnostic indications

**Figure 3.**
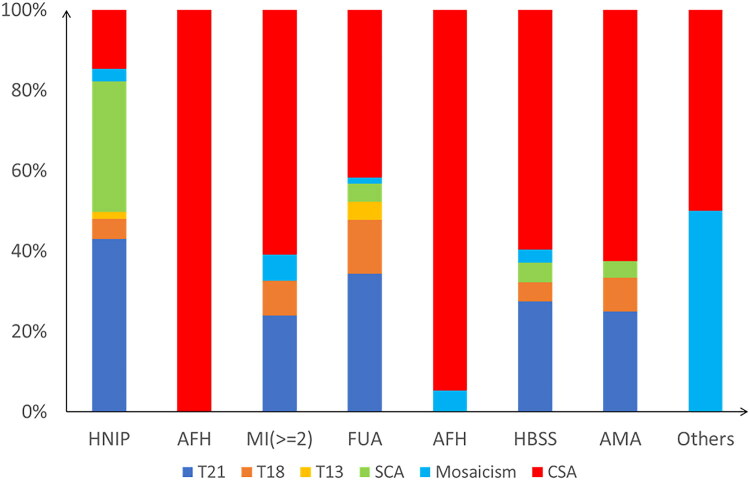
Detection rate ranking of chromosomal abnormalities in 610 patients across various prenatal diagnostic indications from 2014 to 2023. HBSS: high-risk biochemical serological screening; HNIPT: high-risk NIPT; FUA: fetal ultrasound abnormalities; AMA: advanced maternal age; AOH: adverse obstetric history; AFH: adverse family history; MI: multiple indications; T21: Trisomy 21; T18: Trisomy 18; T13: Trisomy 13; SCA: sex chromosome abnormalities; CSA: chromosomal structural abnormalities.

Of the 3,458 amniotic fluid samples, 3,350 routine amniotic fluid cell karyotype analysis, nine culture failure, and a total of 3,341 chromosome karyotype results. Among the different indications for prenatal diagnosis, the high-risk group of NIPT had the highest detection rate (44.40%, 353/795), and the lowest detection rate was in the advanced age group (6.70%, 24/358). Among the 3,458 different indications for prenatal diagnosis, 610 abnormal chromosome karyotypes were detected with a detection rate of 18.25% (610/3,341), with the highest detection rate of trisomy 21(T21) (6.26%, 209/3,341) and the lowest detection rate of trisomy 13(T13) (0.03%, 9/3,341). The proportion of chromosome number abnormalities was higher than that of chromosome structure abnormalities (Table 3 and Figure 3).

**Table 3.** Detection rate ranking of chromosomal abnormalities in 610 patients across various prenatal diagnostic indications.

Rank	Amniocentesis indication	T21	T18	T13	SCA	Mosaicism	CSA	Total
1	HNIPT	152 (19.12%)	18 (2.26%)	6 (0.75%)	115 (14.34%)	11 (1.38%)	52 (6.54%)	354/795 (44.53%)
2	AFH	0 (0.00%)	0 (0.00%)	0 (0.00%)	0 (0.00%)	0 (0.00%)	34 (39.53%)	34/86 (39.53%)
3	MI (≥2)	11 (3.11%)	4 (1.13%)	0 (0.00%)	0 (0.07%)	3 (0.08%)	28 (7.91%)	46/354 (12.99%)
4	FUA	23 (3.47%)	9 (1.36%)	3 (0.45%)	3 (0.45%)	1 (0.15%)	28 (4.22%)	67/663 (10.11%)
5	AOH	0 (0.00%)	0 (0.00%)	0 (0.00%)	0 (0.00%)	1 (0.39%)	18 (7.09%)	19/254 (8.66%)
6	HBSS	17 (2.22%)	3 (0.39%)	0 (0.00%)	3 (0.39%)	2 (0.26%)	37 (4.83%)	62/765 (8.10%)
7	AMA	6 (1.68%)	2 (0.56%)	0 (0.00%)	1 (0.28%)	0 (0.00%)	15 (4.19%)	24/358 (6.70%)
8	Other factors	0 (0.00%)	0 (0.00%)	0 (0.00%)	0 (0.00%)	2 (3.03%)	2 (3.03%)	4/66 (6.06%)
Total		209 (6.26%)	36 (1.08%)	9 (0.27%)	122 (3.65%)	20 (0.60%)	214 (6.41%)	610/3,341 (18.25%)

HNIPT: high-risk NIPT; AFH: adverse family history; MI: multiple indications; FUA: fetal ultrasound abnormalities; AOH: adverse obstetric history; HBSS: high-risk biochemical serological screening; AMA: advanced maternal age.

**Figure 4.**
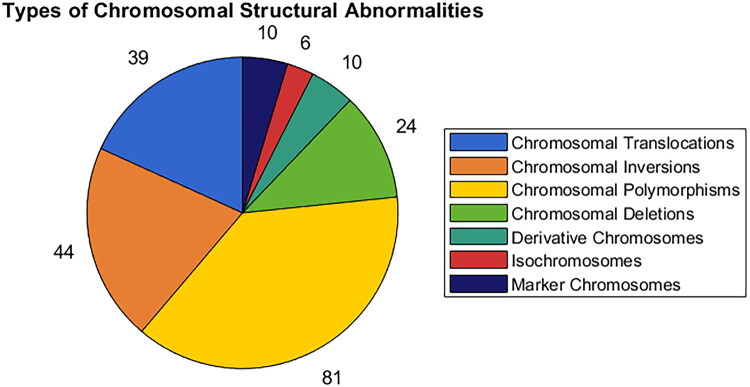
Types of chromosomal structural abnormalities. The pie chart on the left-hand side illustrates the proportional distribution of various components, while the corresponding legend on the right-hand side delineates the nomenclature associated with each chromatic designation.

Among the 610 patients with abnormal chromosomal karyotypes, 214 (35.08%, 214/610) had structural chromosomal abnormalities. Among these, chromosomal translocations comprised 39 cases (6.39%, 39/610), and chromosomal inversions were noted in 44 cases (7.21%, 44/610), with 46,XN,inv(9) being the most frequent, occurring in 30 cases (4.92%, 30/610). Chromosomal polymorphisms were detected in 83 patients (13.61%, 83/610), chromosomal deletions in 24 patients (3.93%, 24/610), derivative chromosomes in 10 patients (1.64%, 10/610), and isochromosomes and marker chromosomes in 6 patients (0.98%, 6/610) and 10 patients (1.64%, 10/610), as shown in Figure 4. Among 610 cases with chromosomal abnormality, 396 had chromosomal numerical abnormality, of which 372 chose elective termination and 24 continued pregnancy. There were 214 cases with chromosomal structural abnormality, including 50 cases of elective termination and 164 cases of continued pregnancy.

In the AFH group, the highest prevalence of chromosomal structural abnormalities was observed (39.53%, 34/86), with all types originating from parental sources. In the HNIPT group, chromosomal deletions were the most prevalent (38.46%, 20/52), followed by chromosomal polymorphisms (17.30%, 9/52). In the AOH group, chromosomal polymorphisms were the most common structural abnormalities (61.11%, 11/18), followed by chromosomal inversions (27.78%, 5/18). In the HBSS group, chromosomal polymorphisms emerged as the most frequent finding (81.08%, 30/37), with chromosomal inversions being the second most common (13.51%, 5/37). In the AMA group, chromosomal polymorphisms constituted the primary structural abnormality (66.67%, 10/15), followed by chromosomal inversions (20%, 3/15), as seen in Table 3.

### Analytical outcomes and PPVs for high-risk NIPT prenatal diagnosis

The detection rate of chromosomal abnormalities in the high-risk NIPT cohort in this study was 44.40%. The PPV was most significant for trisomy 21, reaching 81.72%. The PPVs for different abnormalities within the high-risk NIPT category are detailed in Table 4.

**Table 4.** PPVs For HNIPT invasive prenatal diagnosis.

Rank	HNIPT category	HNIPT cases	Abnormal prenatal diagnosis karyotypes	PPV
1	High risk for T21	186	152	81.72%
2	High risk for SCA	296	115	38.85%
3	High risk for T18	56	18	32.14%
4	High risk for others	221	52	23.53%
5	High risk for T13	39	6	15.38%
Total		795	353	44.40%

HNIPT: high-risk NIPT; PPV: positive predictive value.

### Composition ratio of CNV-seq detection rate

**Figure 5.**
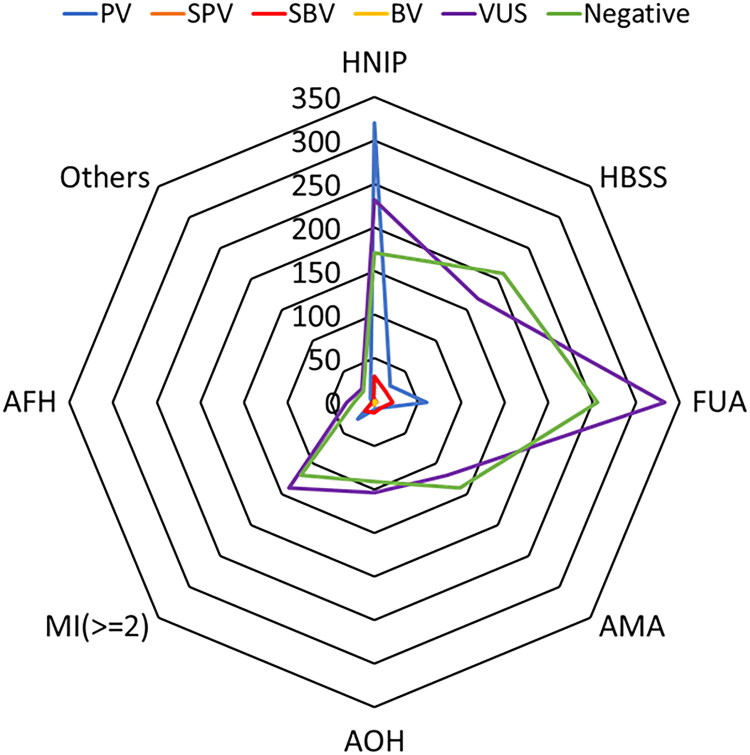
Distribution and composition ratio of CNV-seq results in 2,771 prenatal diagnostic samples. PV: pathogenic variants; SPV: suspected pathogenic variants; SBV: suspected benign variants; BV: benign variants; VUS: variants of uncertain significance.

Among 2,771 different indications for prenatal diagnosis, 460 pathogenic variants(PV) were detected. The highest rate of PV detection was observed in the HNIPT group, at 42.21% (320/758). Conversely, the AOH group had the lowest detection rate of PV at 0.41% (9/222). Variants of uncertain significance (VUS) emerged as the most frequently detected CNV-seq category, accounting for 40.64% (1,126/2,771), while suspected pathogenic variants (SPV) were the least frequently detected, with a total of 20 cases (0.07%, 20/2,771). Comprehensive details are presented in Table 5 and Figure 5.

**Table 5.** Distribution and composition ratio of CNV-seq results in 2,771 prenatal diagnostic samples.

Amniocentesis indication	PV	SPV	SBV	BV	VUS	Negative	Total
HNIPT	320 (42.21%)	4 (0.05%)	30 (3.96%)	1 (0.01%)	232 (30.60%)	171 (22.56%)	758 (27.35%)
HBSS	26 (6.13%)	2 (0.05%)	18 (4.25%)	1 (0.02%)	168 (39.62%)	209 (49.29%)	424 (15.30%)
FUA	60 (8.89%)	3 (0.04%)	21 (3.11%)	2 (0.03%)	333 (49.33%)	256 (37.93%)	675 (24.36%)
AMA	9 (3.25%)	1 (0.04%)	10 (3.61%)	0 (0.00%)	118 (42.60%)	139 (50.18%)	277 (10.00%)
AOH	9 (0.41%)	4 (0.18%)	12 (0.54%)	2 (0.09%)	104 (46.85%)	91 (40.99%)	222 (8.01%)
MI (≥2)	27 (8.91%)	3 (0.10%)	15 (4.95%)	0 (0.00%)	139 (45.87%)	119 (39.27%)	303 (10.93%)
AFH	3 (4.76%)	1 (1.59%)	3 (4.76%)	0 (0.00%)	32 (50.79%)	24 (38.10%)	63 (2.27%)
Other factors	6 (12.24%)	2 (4.08%)	2 (4.08%)	0 (0.00%)	21 (42.86%)	18 (36.73%)	49 (1.77%)
Total	460 (16.60%)	20 (0.07%)	111 (4.01%)	6 (0.02%)	1126 (40.64%)	1009 (36.41%)	2,771

PV: pathogenic variants; SPV: suspected pathogenic variants; SBV: suspected benign variants; BV: benign variants; VUS: variants of uncertain significance; HNIPT: high-risk NIPT; HBSS: high-risk biochemical serological screening; FUA: fetal ultrasound abnormalities; AMA: advanced maternal age; AOH: adverse obstetric history; MI: multiple indications; AFH: adverse family history.

### Karyotyping and CNV-seq abnormality detection rate under different indications for prenatal diagnosis

In HNIPT group, the abnormal detection rate of karyotype analysis was 10.57% (353/3,341), and the detection rate of CNV-seq was 11.55% (320/2,771). In FUA group, the abnormal detection rate of karyotype was 2.01% (67/3,341), and the detection rate of CNV-seq was 2.17% (60/2,771). In HBSS group, the rate of karyotype was 1.86% (62/3,341) and CNV-seq was 0.94% (26/2,771). In AMA group, the rate of karyotype was 0.72% (24/3,341) and CNV-seq was 0.32% (9/2,771). HNIPT showed high abnormal detection rates in both karyotype and CNV-seq tests (Table 6).

**Table 6.** Karyotype and CNV-seq detection rates in each prenatal diagnostic indicator (cases, %).

Amniocentesis indication	Karyotype (3,341)		CNV-seq (2,771)	
	Chromosomal abnormalities and the positive rate	Relevance ratio	Pathogenic variants and the positive rate	Relevance ratio
HNIPT	353/795 (44.40)	10.57	320/758 (42.22)	11.55
HBSS	67/663 (10.11)	2.01	60/675 (8.89)	2.17
FUA	62/765 (8.10)	1.86	26/424 (6.13)	0.94
AMA	46/354 (12.99)	1.38	27/303 (8.91)	0.97
AOH	34/86 (39.53)	1.02	3/63 (4.76)	0.11
MI (≥2)	24/358 (6.70)	0.72	9/277 (3.25)	0.32
AFH	19/254 (8.66)	0.57	9/222 (4.05)	0.32
Other factors	4/66 (6.06)	0.12	6/49 (12.24)	0.22
Total	610/3,341 (18.25)	18.25	460/2,771 (16.60)	16.60

CNV-seq: copy number variation sequencing; HNIPT: high-risk NIPT; HBSS: high-risk biochemical serological screening; FUA: fetal ultrasound abnormalities; AMA: advanced maternal age; AOH: adverse obstetric history; MI: multiple indications; AFH: adverse family history.

### The chromosomal karyotype was normal in 80 cases, while CNV-seq detected pathogenic mutations and pregnancy outcomes

Among 2,731 fetuses exhibiting a normal chromosomal karyotype, CNV-seq detected pathogenic CNV segments in 80 cases. Of these, 63 cases were associated with a pathogenic type manifested through partial deletions; 17 cases resulted from partial duplications; and in 3 cases, two pathogenic segments were identified simultaneously. The most commonly identified pathogenic segment was a partial deletion on 22q11.21 (accounting for 8 out of 80 cases, 8.51%), with deletions ranging from 127.68 kb to 2.90 Mb. A partial deletion in Xp22.31 was the second most frequent (7 out of 80 cases, 7.45%), with deletions between 1.68 and 1.78 Mb, as shown in Table 7. Among the 80 cases, 18 cases chose to induce labor, 56 cases chose to continue pregnancy, of which 1 case had spontaneous abortion at 6 months of pregnancy, one case was born with neonatal lupus accompanied by pneumonia, liver injury, etc., so far, the follow-up has been hospitalized for several times, and the prognosis is relatively poor, and the other 48 cases have been normal after birth follow-up, and 6 cases failed to follow up.

**Table 7.** Ranking of pathogenic variants detected by CNV-seq in 80 patients with a normal chromosomal karyotype.

Rank	Variant type	Number of cases, %
1	Partial deletion on 22q11.21	8 (10.00%)
2	Partial deletion on Xp22.31	7 (8.75%)
3	Partial deletion on 15q11.2	6 (7.50%)
4	Partial deletion on Yq11.223	5 (6.25%)
5	Partial deletion on 7q11.23	3 (3.75%)

CNV-seq: copy number variation sequencing.

### The pathogenic variation of CNV-seq was detected in 108 cases without karyotype detection

Among 108 patients without karyotype analysis (older gestational age), 14 cases (12.96%, 14/108) of CNV-seq were found to have chromosomal pathogenic fragments, of which two cases (1.85%, 2/108) were T21, and 10 cases (9.26%, 10/108) were partially absent. In two cases (1.85%, 2/108), the pathogenic type was partially repeated.

### Detection rate of CNV-seq in combination with karyotype analysis

In this study, among 2,731 samples with normal karyotype, CNV-seq detected pathogenic fragments of CNV in 80 cases, and 14 cases of CNV in 108 samples without karyotype. When CNV-seq was combined with chromosome karyotype, the abnormality detection rate increased to 20.36% (704/3,458), which increased the abnormality detection rate by about 2.11% compared with conventional karyotype technology alone.

## Discussion

The emergence of advanced screening methods, such as NIPT and the application of CNV-seq technology have completely changed the detection process and model in the field of prenatal screening and diagnosis [6]. In recent years, with the adjustment of the fertility policy, the proportion of elderly pregnant women has increased after the opening of the three-child policy [7], the promotion of high-level screening method NIPT and the application of CNV-seq technology have made new changes in the indication composition of prenatal diagnosis. We retrospectively analyzed the trend of different prenatal diagnostic indicators in Heilongjiang Province in the past 10 years (2014–2023), as well as the analysis results of amniotic fluid karyotype and CNV-seq, and discussed its clinical application value.

In China, in recent years, high risk of biochemical serological screening and abnormal ultrasound are the main indications for prenatal diagnosis. However, as the cost of NIPT decreases and clinical use increases, the proportion of prenatal diagnoses resulting from HNIPT is steadily increasing. From 2014 to 2023, HNIPT was the most common indicator of prenatal diagnosis in Heilongjiang Province (23.13%, 800/3,458), which emphasizes the dominant role of NIPT as the main method of prenatal screening [8]. However, AMA was only the fourth in the list of prenatal diagnostic indicators in our province (359/3,458, 10.38%), which was significantly different from studies in other regions [9]. This difference may be due to the relative economic development of our province and the small birth population. In addition, after NITP was first administered to older women [10], the number of invasive prenatal diagnoses decreased. Guidelines emphasize that invasive prenatal diagnostic testing is primarily based on positive screening tests and not solely on maternal age [11].

In our study, the proportion of HBSS decreased year by year from 2014 to 2023, and only accounted for 5.18% in 2022. In addition, the proportion of HNIPT has been increasing year by year since 2014, reaching 27.73% in 2017 and maintaining a high proportion since then. The proportion of AMA fluctuated, but showed an increasing trend in the past three years. Overall, HBSS, HNIPT, and FUA are still the main prenatal diagnostic indications in recent years. In China, prenatal screening is not completely mandatory, but it is part of the national recommended maternal and child health service program, and high-risk pregnant women are encouraged to participate actively. As two different screening methods, biochemical serological screening and NITP can be selected. Biochemical serological screening will still be the main primary screening method in primary hospitals because of its low cost and relatively simple detection technology. Although NIPT is expensive but accurate, some families take the initiative to choose to replace biochemical serological screening. According to relevant guidelines in China, such as the Measures for the Management of Prenatal Diagnosis Techniques, it is suggested that pregnant women over 35 years of age belong to a high-risk group, and invasive prenatal diagnosis, such as amniocentesis, is recommended instead of serological screening. However, in actual life, many pregnant women will still do NIPT as a preliminary screening, and then do amniocentesis if the result is high risk. In addition, older pregnant women are concerned about invasive prenatal diagnosis and may prefer NIPT. From the regional point of view, prenatal diagnosis center of Heilongjiang Province is a third class A hospital, and pregnant women are more inclined to choose NIPT. Invasive prenatal diagnosis requires referral to a qualified prenatal diagnosis facility, and more pregnant women with HBSS tend to preferentially choose NIPT due to limited medical resources and difficulties in the field. The above reasons increase the composition of high risk of NIPT.

In our research, the group classified as HNIPT exhibited the highest chromosomal abnormality detection rate at 44.53% (354/795), establishing it as the category with the most detected abnormal karyotypes. NIPT is especially recognized for its sensitivity in identifying abnormal numbers of chromosomal aneuploidies [12]. Within the HNIPT group in our study, the PPV for T21 was the highest at 81.72%, followed by SCA at 38.85% and T18 at 32.14%. International research has indicated that NIPT technology has contributed to a >60% reduction in invasive procedures among patients with high-risk prenatal diagnostic factors while maintaining a comparable rate of chromosomal disease detection [13]. Nonetheless, given NIPT’s inability to detect a comprehensive range of chromosomal abnormalities, combining maternal age, serological screening, prenatal ultrasound, and NIPT is recommended for comprehensive prenatal evaluation. This combined approach enhances the likelihood of detecting a broader spectrum of abnormalities [14].

In this study, FUA was found to be the leading prenatal diagnostic indication in 2020, indicating that advances in ultrasound technology and improved diagnostic skills can help in the timely detection of subtle or complex fetal structural abnormalities. Prenatal ultrasound, as a noninvasive screening method, is favored because patient ultrasound equipment resolution has made fetal ultrasound abnormalities a primary prenatal diagnostic indicator in our area. In our research, the FUA group exhibited an abnormal karyotype detection rate of 21.66% (749/3,458), which was higher than the detection rate of 18.2% found in other domestic hospitals [15]. This discrepancy may be due to regional economic development, the extent of the sample size in the study, and the fact that our hospital receives a large number of referrals for prenatal ultrasound abnormalities from throughout the province.

Based on high-throughput sequencing technology, CNV-seq can sequence whole genome DNA and accurately detect chromosome copy number variation with a resolution of up to kb. CNV-seq can identify small fragments of pathogenic variation that cannot be identified by traditional karyotype analysis, and can meet different clinical needs by changing the sequencing depth [16]. Due to considerations, such as testing costs and the acceptance of testing technologies, widespread implementation of CNV-seq testing in our province’s prenatal clinical practice commenced only in 2017. From 2017 to 2023, prenatal samples from 2,771 women were subjected to CNV-seq, resulting in a pathogenic CNV detection rate of 16.60% (460/2,771). This rate is higher than that reported in other domestic studies [17]. The HNIPT group showed the highest percentage of pathogenic CNV, 42.21% (320/758), highlighting the sensitivity and specificity of NIPT in accurately screening for fetal pathogenic CNV [18]. In addition, in 460 cases of pathogenic CNV, the proportion of FUA (8.89%, 60/460) was the third in line. In particular, when ultrasound indicates that heart abnormalities are combined with other organ malformations, CNV-seq detection can not only improve the detection rate [19], but also may find the cause of ultrasound abnormalities in fetuses with normal or non-karyotyped karyotyping [20].

In this study, among 2,731 samples with normal karyotype, 80 cases of CNV-seq were found to have CNV pathogenic variation, while 14 cases of CNV pathogenic variation were also detected in 108 samples without karyotype. Compared with the detection rate of single karyotype, the detection rate of CNV-SEQ combined with karyotype could increase to 20.36%. The anomaly detection rate was increased by 2.11%. The advantage of conventional karyotyping is the detection of structural anomalies, such as balanced translocation and inversion, but CNV-seq can detect minor copy number variations, such as DiGeorge syndrome and Williams syndrome. The combined application of the two can improve the abnormal detection rate and provide more comprehensive information for clinical diagnosis. At the same time, CNV-seq can further determine the size, location, and genetic composition of abnormal fragments, help doctors better assess the prognosis of the fetus, and provide more accurate genetic counseling and fertility guidance for pregnant women and their families. It is important to note that the constant updating of CNV databases, as well as a deeper understanding of CNV variants and associated diseases, also brings a level of uncertainty that complicates counseling and clinical decision making for potentially pathogenic CNVs cases. The nature, size, and genetic factors of CNV variation will affect its pathogenicity [21]. Integrating parental verification and understanding familial genetic information is essential to thoroughly assess its clinical relevance.

Although the cost of direct testing for CNV-seq is high, it is relatively cost-effective from the perspective of the entire healthcare system and society, considering its significant benefits in improving diagnostic accuracy, guiding precision treatment, genetic counseling, and promoting medical research. Especially for the detection of some high-risk groups and difficult cases, its benefits are more prominent. With the increasing maturity of the technology, the continuous expansion of the promotion scope, and the gradual reduction of the cost, we believe that CNV-seq technology is expected to become the first-line technology of prenatal diagnostic testing in the future.

## Conclusions

Our study is the first to summarize trends in the composition of 3458 prenatal diagnostic indicators from a single central source in Heilongjiang Province over the past 10 years. The detection of chromosomal abnormalities was summarized, and the role of the combination of karyotype and CNV-seq in prenatal diagnosis and the effectiveness of prevention of BDs were analyzed. In recent years, with the promotion of NIPT and the improvement of prenatal ultrasound technology, HNIPT and FUA have gradually become the main prenatal diagnostic indications in our province. In summary, karyotype analysis combined with CNV-seq technology can increase the detection rate of chromosome abnormalities to 21.68%. Our study recommends the use of the combined CNV-seq technique for all prenatal diagnoses. At the same time, it provides a very effective data reference for our province to further standardize the service process of prenatal screening of high-risk groups and formulate livelihood policies to prevent BDs.

## Data Availability

The CNV-seq raw data for the participants are not publicly available due to privacy and ethical restrictions. Other researchers and readers can access these data by requesting from the corresponding author privately.
